# Supplementary material for: Translation and cultural adaptation of MedStopper®—A web-based decision aid for deprescribing in older adults: A protocol
Source: PLoS One. 2023 Apr 20;18(4):e0284464. doi: 10.1371/journal.pone.0284464 (PMC10118160; doi:10.1371/journal.pone.0284464)
Supplement: S1 Timeline — (PDF) [file pone.0284464.s001.pdf]

PLOS ONE

REGISTERED REPORT PROTOCOL

Translation and cultural adaptation of a web-based decision aid for deprescribing in older adults: A protocol

Luís Monteiro[1,2]*, **Sofia Baptista**[1,3], **Inês Ribeiro-Vaz**[1,3,4], **James McCormack**[5], **Cristiano Matos**[6], **Andreia Teixeira**[1,3,7], **Matilde Monteiro-Soares**[1,3], **Carlos Martins**[1,3]

1 CINTESIS—Centre for Health Technology and Services Research, Faculty of Medicine, University of Porto, Porto, Portugal; **2** USF Esgueira +, Aveiro, Portugal; **3** MEDCIDS—Department of Community Medicine, Information and Decision in Health, Faculty of Medicine, University of Porto, Porto, Portugal; **4** Porto Pharmacovigilance Centre, Faculty of Medicine, University of Porto, Porto, Portugal; **5** Faculty of Pharmaceutical Sciences, University British Columbia, Canada **6** Escola Superior de Tecnologia da Saúde de Coimbra, Instituto Politécnico de Coimbra, Coimbra, Portugal; **7** IPVC—Instituto Politécnico de Viana do Castelo, Viana do Castelo, Portugal;

* luismonteiro.net@gmail.com

Check for updates

This is a Registered Report and may have an associated publication; please check the article page on the journal site for any related articles.

OPEN ACCESS

Citation: Monteiro L, Baptista S, Ribeiro-Vaz I, McCormack J, Matos C, Teixeira A, Monteiro-Soares M, Martins, C (2023) Translation and cultural adaptation of a web-based decision aid for deprescribing in older adults: A protocol. PLoS ONE xx(xx): xxxxxxxxx. https://doi.org/10.1371/journal.ponexxxxxxxxx.

Editor: Name

Received: date

Accepted: date

Published: date

Copyright: © 2023 Monteiro et al. This is an open access article distributed under the terms of the Creative Commons Attribution License, which permits unrestricted use, distribution, and reproduction in any medium, provided the original author and source are credited.

Data Availability Statement: All relevant data from this study will be made available upon study completion.

Funding: The authors received no specific funding for this work.

Competing interests: The authors have declared that no competing interests exist.

Abbreviations: PIMs, potentially inappropriate medications; STOPP, Screening Tool of Older People's Prescriptions; START, Screening Tool to Alert to Right Treatment; ECDC, European Centre for Disease Prevention and Control's

Supporting Information

Appendix S1 Timeline

	2023				2024				2025			
**Tasks/Trimester**	**1**	**2**	**3**	**4**	**1**	**2**	**3**	**4**	**1**	**2**	**3**	**4**
**Step 1:** Selection of materials and process coordinators				■								
**Step 2:** Early review					■							
**Step 3:** Translation and back-translation						■						
**Step 4:** Comprehension testing							■	■				
**Step 5:** Proofreading									■			
